# Estimating Community Incidence of *Salmonella, Campylobacter*, and Shiga Toxin–producing *Escherichia coli* Infections, Australia

**DOI:** 10.3201/eid1410.071042

**Published:** 2008-10

**Authors:** Gillian Hall, Keflemariam Yohannes, Jane Raupach, Niels Becker, Martyn Kirk

**Affiliations:** Australian National University, Acton, Australian Capital Territory, Australia (G. Hall); Department of Health and Ageing, Canberra, Australia (K. Yohannes, M. Kirk,); Department of Health, Adelaide, South Australia, Australia (J. Raupach); National Centre for Epidemiology and Population Health (G. Hall, N. Becker)

**Keywords:** Australia, surveillance, salmonellosis, campylobacteriosis, Shiga toxin-like Escherichia coli (STEC), notification-fraction, multipliers, credible interval, incidence, research

## Abstract

To estimate multipliers linking surveillance of salmonellosis, campylobacteriosis, and Shiga toxin–producing *Escherichia coli* (STEC) infections to community incidence, we used data from a gastroenteritis survey and other sources. Multipliers for severe (bloody stool/long duration) and milder cases were estimated from the component probabilities of doctor visit, stool test, sensitivity of laboratory test, and reporting to surveillance system. Pathogens were classified by the same severity criteria and appropriate multipliers applied. Precision of estimates was quantified by using simulation techniques to construct 95% credible intervals (CrIs). The multiplier for salmonellosis was estimated at 7 (95% CrI 4–16), for campylobacteriosis at 10 (95% CrI 7–22), and for STEC at 8 (95% CrI 3–75). Australian annual community incidence rates per 100,000 population were estimated as 262 (95% CrI 150–624), 1,184 (95% CrI 756–2,670), and 23 (95% CrI 13–54), respectively. Estimation of multipliers allows assessment of the true effects of these diseases and better understanding of public health surveillance.

## Abstract 2

Estimated multipliers that linked surveillance of foodborne diseases with community incidence showed a high prevalence of these diseases.

The primary aims of laboratory-based surveillance of *Salmonella, Campylobacter,* and Shiga toxin–producing *Escherichia coli* (STEC) infections in industrialized countries are to detect outbreaks and to monitor changes in incidence over time. For laboratory-based surveillance, a person with diarrhea must visit a doctor, have an appropriate stool sample transported to the laboratory correctly, have a positive laboratory test for a notifiable disease, and have this result reported to the surveillance system. Because many persons do not visit a doctor or have a stool sample taken when they have diarrhea, surveillance data do not capture all disease and represent only a fraction of disease occurring in the community. However, knowledge of the absolute number of cases in the community would be extremely useful for setting public health policy and estimating the cost of illness.

Studies that have estimated community incidence have used various methods. These include: capture-recapture (*1*), Delphi or expert consensus (*2*), outbreak reports (*3*,*4*), community-based cohort studies of diarrheal disease (*5*), and estimation of multipliers of surveillance data by using additional data (*6**)*. Although cohort studies are the most direct way of estimating diarrheal illness in the community, they are expensive and are subject to limitations for estimating the incidence of pathogen-specific infections because of the small numbers of cases (*7*,*8*).

This article describes how we estimated multipliers to apply to laboratory surveillance data to estimate community incidence, including estimation of precision. We used data collected in a survey of gastroenteritis and data from case-control studies and from quality assurance of laboratory testing to estimate the major component parts of the multipliers. The major components are the proportion of case-patients who visit a doctor, the proportion of these patients who have a stool sample sent to the laboratory for testing, the sensitivity of the laboratory test to correctly identify a pathogen, and the proportion of positive results that are reported to surveillance by laboratories. These component parts were then used to estimate the overall multipliers, and the precision of these estimates was determined.

In 2005, a total of 7,720 cases of salmonellosis, 15,313 cases of campylobacteriosis, and 85 cases of STEC infection were reported to the Australian National Notifiable Diseases Surveillance System. The specific objective of this study was to estimate the multipliers to apply to *Salmonella, Campylobacter,* and STEC infections reported to national surveillance and to estimate the community incidence of these conditions in Australia.

## Methods

### Overview

The fraction of community cases reported to surveillance was derived from the probability of a case-patient in the community visiting a doctor, having a stool sample taken, having a positive laboratory test, and having the case reported to surveillance (Figure). At every step a proportion of cases from the previous step are lost, resulting in only a fraction of cases being reported (the notification fraction).

**Figure:**
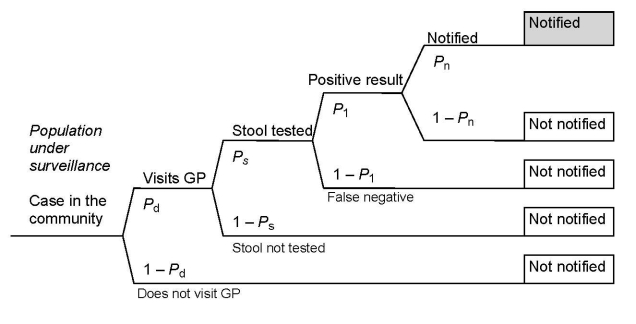
Sequential steps for notification to a surveillance system. The probability of progressing in the sequential steps in the surveillance system is represented by *P.* GP, general practitioner.

The notification fraction (NF) is equal to the product of the component probabilities; that is, NF = *P_d_ × P_s_ × P_l_ × P_n_* (Figure), where *P*_d_ = probability that a case-patient in the community visits a doctor, *P*_s_ = probability that a case-patient seen by a doctor has a stool sample taken, *P*_l_ = probability that a laboratory correctly identifies the pathogen in the stool sample, and *P*_n_ = probability that a positive result is reported to health authorities. The reciprocal of the notification fraction is the multiplier M (M = 1/NF).

The probabilities of visiting a doctor and of having stool tested are likely to be greater when illness is more severe. We therefore assessed the influence of different symptoms on the likelihood of gastroenteritis patients visiting a doctor and having a stool sample taken. Influential symptoms were used to classify gastroenteritis cases by severity, and multipliers were calculated for the different severity categories. The 3 infections of interest—salmonellosis, campylobacterosis, and STEC infection—were then classified by using the same categories so that appropriate multipliers could be applied, based on the severity of the particular illness. Uncertainty in the estimates was estimated by using simulation techniques to derive a 95% credible interval (95% CrI), akin to methods used in Bayesian estimations.

### Data Sources

We derived our estimates from data already available in Australia on diarrhea, salmonellosis, campylobacteriosis, and STEC infection (Table 1).

**Table 1:** Data sources used to assess under-reporting of *Salmonella*, *Campylobacter*, and STEC infections, Australia, 2001–2005*

Information	Data sources
Symptoms that predicted visiting a doctor and having stool tested (“predictor symptoms”) used to adjust calculations for severity of illness	Australian National Gastroenteritis Survey (NGS) conducted across Australia during 2001 and 2002 (*9*)
Probability of a case-patient in the community visiting a doctor	NGS
Probability of a case-patient seen by a doctor having stool tested	NGS and unpublished reports of 2 surveys of GP treatment and management practices for gastroenteritis in 2003/2004 and 2005 in 2 Australian states (*10*,*11*)
Probability of correctly identifying *Salmonella* and *Campylobacter* in stool samples by laboratories	Royal College of Pathologists Australasia, Quality Assurance Programs Pty Limited, Microbiology QAP Results, 2001 (*12*)
Probability of a positive result being reported to health authorities	Discussions with OzFoodNet epidemiologists
Symptom profiles for reported cases of salmonellosis	Unpublished case-control study data from the Hunter Public Health Unit, NSW Australia (1997–2000), and OzFoodNet sites (2000–2003)
Symptom profiles on reported cases of campylobacteriosis	Unpublished case control study data from the Hunter Public Health Unit, NSW Australia (1997–2000), and OzFoodNet sites (2000–2003)
Information on reported cases of STEC, and laboratory sensitivity of detecting STEC from fecal samples	Unpublished data from OzFoodNet study on STEC in South Australia, 2003–2005
Number of notifications of campylobacteriosis, salmonellosis, and STEC infection.	National Notifiable Diseases Surveillance System (*13*)
Australian midyear population for 2005	Australian Bureau of Statistics (*14*)

*STEC, Shiga toxin–-producing *Escherichia coli;* GP, general practitioner; QAP, quality assurance program. Further details of how data were used are shown in the Technical Appendix

#### Estimation of Symptom-specific Probabilities for Visiting a Doctor and Having a Stool Test (*P_d_* and *P_s_*)

The component probabilities *P_d_* and *P_s_* were estimated from the National Gastroenteritis Survey (NGS) conducted in 2002. The telephone survey, using a random stratified sample from all states in Australia, included a total sample of 6,087 persons who were asked about diarrheal illness they had experienced in the previous 4 weeks. Respondents with chronic illness with diarrhea were not counted as case-patients unless they identified their symptoms as different than usual. Case-patients were asked details about their illness and days of duration of symptoms (*9*). The case definition of diarrheal illness was the following: at least 3 loose stools in 24 hours (excluding those persons who report a noninfectious cause of their diarrhea because of pregnancy, alcohol use, chronic illness, or medications) and duration <28 days.

#### Adjusting Component Probabilities for Severity of Illness

To calculate improved pathogen-specific estimates rather than estimates of underreporting of all gastroenteritis, we estimated multipliers from NGS for gastroenteritis of varying severity. The proportion of cases in corresponding severity categories was then estimated for the 3 different pathogens by using other data from case-control studies (Table 1). The appropriate multipliers could then be applied according to the average severity of illness.

Symptoms of severity that predicted whether the person with gastroenteritis visited a doctor were identified from NGS by using multivariable logistic regression. The following conditions were evaluated: duration of illness, cramps, vomiting, nausea, blood in the stool, headache, respiratory symptoms (coughing, sneezing, sore throat), fever, body aches, loss of appetite, and stiff neck. Age and sex of the patients were also considered. A p value <0.05 was considered statistically significant.

Because the number of case-patients who had stool tests was small in the gastroenteritis survey (n = 15), univariate regression was used to identify severity symptoms that predicted whether a doctor ordered a stool test (statistical significance p<0.1). Additional information from a random sample of general practitioners (GPs) from 2 states of Australia on the treatment of diarrheal disease was used to identify severity symptoms that prompted doctors to order a stool test (*10**,**11*). Data were stratified by symptom categories that were statistically significant determinants for both visiting a doctor and having a stool test ordered. Probabilities were calculated for case-patients in each symptom category of visiting a doctor and having stool tested.

#### Pathogen-specific Symptom Profiles

The severity symptom profiles of salmonellosis and campylobacterosis were developed from OzFoodNet national case-control studies based on 396 and 1,087 reported cases, respectively (Table 1). The STEC symptom profile was based on a case-control study of 34 cases in South Australia. The proportion of case-patients who experienced the predictor symptoms (i.e., symptoms that were found to predict visiting a doctor and a doctor’s ordering of stool tests) was calculated. From this calculation, the probabilities of visiting a doctor and having a stool test were estimated for each pathogen, weighted by severity of illness.

#### Sensitivity of Laboratory Testing

All fecal specimens are routinely tested for *Salmonella* and *Campylobacter* spp. in Australia. The sensitivities (*P_l_*) of laboratory tests to detect *Salmonella* and *Campylobacter* spp. were obtained from quality assurance data from ≈250 laboratories across the country (*12*). This sensitivity testing is based on inoculated samples that are transported to laboratories from a central source. For STEC, data were from the state of South Australia, where enhanced surveillance exists for this pathogen. Laboratories routinely forward all stool samples with macroscopic blood to a reference laboratory, which tests for STEC by using molecular methods to detect genes encoding for production of Shiga toxin 1 and Shiga toxin 2 (*15*).

#### Reporting to Surveillance Network

The completeness of mandatory reporting to health departments was discussed with state epidemiologists involved in OzFoodNet, Australia’s foodborne disease surveillance network. Based on these discussions, we concluded that mandatory reporting to health departments has been virtually complete for these pathogens for several years preceding 2005 because of the widespread use of computerized reporting practices in Australian laboratories. This information was used to estimate the probability for a positive result to be reported to health authorities (*P*_n_). Formal validation studies on completeness of reporting were not available.

#### Uncertainty and 95% CrIs

Simulation techniques were used to quantify the degree of uncertainty of the underreporting factors. The uncertainty in our knowledge about each of *P_d_*, *P_s_*, and *P_l_* was quantified in terms of a normal distribution as this is a simple technique easily applied in different situations. However, because some data may be best simulated by using other distributions (such as probabilities close to 1 or 0 and other nonsymmetric distributions), we compared the output from simulations that used normal distributions with simulations that used other distributions as indicated (Technical Appendix). The different distributions had minimal influence on the outcomes, and therefore all simulations were conducted as normal distributions.

The mean and standard deviation were allocated to describe the simulations when various data sources were used. The mean and standard deviation of the probability of visiting a doctor (*P_d_)* were estimated from the gastroenteritis survey data (*9*). The mean and standard deviation of the probability of stool ordering (*P_s_*) by GPs were quantified by averaging results from the GP surveys conducted in the states of South Australia and Victoria (*10**,**11*). The mean and standard deviation of the sensitivity of stool testing (*P_l_*) were quantified by using quality assurance results from the 2001 report (*12*). If a simulation produced a few negative values, these were treated as missing data. For each pathogen, a distribution was similarly determined for the symptom profiles that described the proportion of cases in each of 6 severity categories by using data from the case-control studies as shown in Table 1.

For each pathogen, 1,000 observations were simulated from each of the distributions and were used to calculate 1,000 estimates of the multipliers. The range between the 2.5 and 97.5 percentiles of this empirical distribution was quoted as a 95% CrI for the final estimates of the multipliers. The parameters used in the simulations are shown in the Technical Appendix tables. The software package SPSS was used for simulations (*16*).

### Calculation of Community Incidence

The number of national notifications for each pathogen from 2000 through 2004 was used to estimate the mean and standard deviation of a normal distribution for the yearly notifications (Table 2). Data were adjusted for total population coverage for *Campylobacter* spp. because 1 state (New South Wales) does not report this pathogen. STEC estimates were based on information from 1 state (South Australia), so South Australian estimates for STEC were then extrapolated to the national population.

**Table 2:** Number of notifications in Australia each year for salmonellosis, campylobacteriosis, and STEC infections, 2000–2004*

Data	*Salmonella* infections	*Campylobacter* infections (all states except NSW)†	STEC infections in SA‡
Year			
2000	6,196	13,665	–
2001	7,047	16,123	27
2002	7,696	14,740	39
2003	7,017	15,369	37
2004	7,829	15,622	30
Mean (SD)	7,157 (651)	15,104 (946)	33.3 (5.67)
Median	7,047	15,369	34
Percentiles: 2.5, 97.5	6,278, 7,816	13,773, 16,073	27, 39

*STEC, Shiga toxin–producing *Escherichia coli*; NSW, New South Wales; SA, South Australia. †67% of population only; adjust for population of Australia by multiplying by 1.5. ‡7.5% of population only; adjust for population of Australia by multiplying by 13.3.

One thousand simulated values from these distributions were then multiplied by the 1,000 simulated values of the pathogen-specific multipliers to obtain 1,000 simulated annual incidences in the community. The median and the 95% CrI from this empirical distribution were estimated as above.

## Results

### Estimation of Symptom-specific Probabilities for Visiting a Doctor and Having a Stool Test

Among respondents of the NGS, 374 persons had diarrhea and met our case definition. Of these case-patients, 75 (20%) visited a doctor for treatment, and for 15 (20%) of 75, a stool test was ordered.

Duration of illness was the most important statistically significant symptom that increased both the probability of visiting a doctor and of having a stool sample taken. The odds of visiting a doctor doubled with each day of duration (p<0.001), and the odds of having a stool test among those who visited a doctor was 1.5 (p = 0.005) for each day of duration. Blood in stool was identified as a strong predictor for a doctor to order a stool test in both of the GP surveys, with 80% and 91%, respectively, of GPs in the 2 surveys “always or nearly always” ordering a stool test if blood was present (*10*,*11*). In the gastroenteritis survey, the presence of blood in the stool did not influence whether a person with gastroenteritis visited a doctor (odds ratio [OR] = 1.5, p = 0.55) but did influence whether a doctor ordered a stool test (OR = 9, p = 0.08). Fever was also statistically significantly associated with visiting a doctor. All other variables were not significant.

Since duration of illness was identified as influential on both the patient’s visiting a doctor and on whether a stool test was ordered, and because blood in stool was also very influential on whether a doctor ordered a stool test, 6 severity categories were created: 3 duration categories (1–2 days, 3–4 days, >5 days) for those with blood in stool, and the same duration categories for those without blood in stool. The component probabilities *P_d_* and *P_s_* were then calculated separately for each severity category as shown in Table 3. Further data details are shown in the Technical Appendix.

**Table 3:** Probabilities and underreporting factors for each category of duration of diarrhea by blood in stool, for salmonellosis, campylobacteriosis, and STEC infections*

Condition/predictor symptoms	Probability of:				Probability for a case to be reported† (95% CrI)	Multiplier‡ (95% CrI)
	(a) Case-patient visiting a doctor (95% CrI)	(b) Stool being tested (95% CrI)	(c) Positive stool results (95% CrI)	(d) Notification by laboratory		
Salmonellosis						
With blood						
1–2 d	0.10 (0.07–0.14)	0.85 (0.72–0.98)	0.98 (0.95–1.00)	1.00	0.09 (0.06–0.12)	11.39 (8.49–16.36)
3–4 d	0.43 (0.31–0.54)	0.85 (0.72–0.98)	0.98 (0.95–1.00)	1.00	0.36 (0.25–0.46)	2.82 (2.17–3.98)
>5 d	0.67 (0.46–0.88)	0.85 (0.72–0.98)	0.98 (0.95–1.00)	1.00	0.55 (0.368–0.75)	1.81 (1.33–2.72)
Without blood						
1–2 d	0.10 (0.07–0.14)	0.07 (0.02–0.02)	0.98 (0.95–1.00)	1.00	0.01 (0.003–0.01)	143.29 (83.30–371.0)
3–4 d	0.43 (0.31–0.54)	0.19 (0.071–0.36)	0.98 (0.95–1.00)	1.00	0.08 (0.010–0.16)	13.06 (6.37–67.83)
>5 d	0.67 (0.46–0.88)	0.40(0.133–0.67)	0.98 (0.95–1.00)	1.00	0.25 (0.075–0.48)	3.93 (2.10–11.92)
Campylobacteriosis						
With blood						
1–2 d	0.10 (0.07–0.14)	0.85 (0.72–0.98)	0.90 (0.85–0.95)	1.00	0.08 (0.056–0.11)	12.40 (9.16–17.82)
3–4 d	0.43 (0.31–0.54)	0.85 (0.72–0.98)	0.90 (0.85–0.95)	1.00	0.33 (0.231–0.43)	3.06 (2.32–4.33)
>5 d	0.67 (0.46–0.88)	0.85 (0.72–0.98)	0.90 (0.85–0.95)	1.00	0.51 (0.339– 0.70)	1.97 (1.42–2.95)
Without blood						
1–2 d	0.10 (0.07–0.14)	0.069 (0.02– 0.12)	0.90 (0.85–0.95)	1.00	0.01 (0.002–0.01)	154.17 (89.31–397.59)
3–4 d	0.43 (0.31–0.54)	0.185 (0.071–0.36)	0.90 (0.85–0.95)	1.00	0.07 (0.009–0.15)	14.15 (6.80–73.32)
>5 d	0.67 (0.46–0.88)	0.400 (0.133–0.67)	0.90 (0.85–0.95)	1.00	0.24 (0.068–0.44)	4.25 (2.25–13.36)
STEC in South Australia						
With blood						
1–2 d	0.10 (0.07–0.14)	0.85 (0.72–0.98)	0.88 (0.83–0.93)	1.00	0.08 (0.0.05–0.11)	13.02 (9.50–18.37)
3–4 d	0.43 (0.31–0.54)	0.85 (0.72–0.98)	0.88 (0.83–0.93)	1.00	0.32 (0.22–0.42)	3.13 (2.36–4.45)
>5 d	0.67 (0.46–0.88)	0.85 (0.72–0.98)	0.88 (0.83–0.93)	1.00	0.50 (0.33–0.68)	2.02 (1.47–3.04)
Without blood						
1–2 d	0.10 (0.07–0.14)	0.07 (0.02–0.12)	0.88 (0.83–0.93)	1.00	0.01 (0.001–0.02)	157.18 (61.67–218.75)
3–4 d	0.43 (0.31–0.54)	0.19 (0.071–0.36)	0.88 (0.83–0.93)	1.00	0.07 (0.01–0.14)	14.35 (7.38–64.34)
>5 d	0.67 (0.46–0.88)	0.40 (0.133–0.67)	0.88 (0.83–0.93)	1.00	0.23 (0.07–0.44)	4.31 (2.27–13.44)

*STEC, Shiga toxin–producing *Esherichia coli*; CrI, credible interval; NF, notification factor. †NF, product of a × b × c × d. ‡Inverse of NF.

Because blood in stool did not predict patient’s health-seeking behavior in the NGS, the estimates of probability of visiting a doctor by duration were made on the whole sample and then the same probabilities were applied to the categories of “with blood” and “without blood” in stool. The probability of visiting a doctor increased from 0.1 to 0.67 as duration increased from 1–2 days to >5 days. Based on the surveys of GPs, we estimated that among patients with bloody diarrhea who visited a doctor, the probability of having a stool test was 0.85, regardless of the duration of their diarrhea. For those without blood in stool, the probability of a stool test increased from ≈0.1 to 0.4 as duration of illness increased.

### Laboratory Testing

Quality assurance testing in 2001 showed that 100% of 254 laboratories correctly detected *Salmonella* spp. from 1 batch of fecal samples, and 233 (95%) of 246 laboratories identified it correctly from a second batch. Of 257 laboratories, 220 (86%) correctly detected *Campylobacter* spp.from 1 batch of fecal samples, and 232 (95%) of 244 laboratories detected it from a second batch (*12*). The probability for correct identification of *Salmonella* spp. by laboratories was therefore estimated to be 0.98 (95% CrI 0.95–1.00) and for identification of *Campylobacter* spp., 0.90 (95% CrI 0.85–0.95).

The probability for correct STEC identification by a laboratory PCR was estimated to be 0.98 (95% CrI 0.95–1.00) (*17*). Macroscopic blood is the major reason for conducting a laboratory test to identity STEC in South Australia. Among those persons with STEC infection, 0.90 (95% CrI 0.85–0.95) of their stools are estimated to have macroscopic blood (*18*). The proportion of stools containing STEC that are identified by laboratories in South Australia was therefore estimated as the product of 0.90 *(*95% CrI; 0.85–0.95) and 0.98 *(*95% CrI 0.95–1.00) to give 0.88 *(*95% CrI; 0.83–0.93).

### Pathogen-specific Multipliers

Underreporting varied considerably by severity of illness, with reporting ranging from ≈l in 2 cases for “severe illness” cases with blood in the stool and long duration, to ≈1 in 150 for “mild illnesses” without blood and shorter duration (Table 3). For every 100 cases reported, the number in each of the 6 severity categories is shown for each pathogen in Table 3. Long duration is common; 83% of salmonellosis cases, 77% of campylobacterosis cases, and 74% of STEC cases lasted at least 5 days. Blood in stool was reported for 50%, 44%, and 86% of salmonellosis, campylobacteriosis, and STEC cases, respectively. The severity-weighted estimates of underreporting for salmonellosis, campylobacteriosis, and STEC infection are shown in Table 4. For every 100 notifications, an estimated 695 (95% CrI 399–1,643) cases of salmonellosis occurred in the community and 1,001 (95% CrI 664–2,251) cases of campylobacteriosis. In South Australia, for every 100 notifications of STEC, an estimated 815 cases (95% CrI 330–7,514) occurred in the community.

**Table 4:** Severity-specific underreporting for salmonellosis, campylobacteriosis, and STEC infections*

Condition/severity category	Symptom multiplier† (95% CrI)	No. reported cases in severity category, in hundreds (95% CrI)	No. cases in the community for every 100 reported‡ (95% CrI)
Salmonellosis			
With blood			
1–2 d	11.39 (8.49–16.36)	1 (0–3)	12.7 (0.8–32.1)
3–4 d	2.82 (2.17–3.98)	7 (5–10)	19.9 (12.6–30.9)
> 5 d	1.81 (1.33–2.72)	42 (37–47)	76.6 (54.3–116.0)
Without blood			
1–2 d	143.29 (83.30–371.0)	2 (1–4)	282.6 (50.4–870.3)
3–4 d	13.06 (6.37–67.83)	7 (5–10)	91.8 (40.3–533.5)
>5 d	3.93 (2.10–11.92)	41 (36–46)	160.8 (85.8–513.8)
Overall		100	695 (399–1,643)
Campylobacteriosis			
With blood			
1–2 d	12.40 (9.16–17.82)	2(1–3)	24.8 (16.3–38.6)
3–4 d	3.06 (2.32–4.33)	8(6, 10)	24.3 (15.8–36.9)
>5 d	1.97 (1.42–2.95)	34 (31, 37)	67.92 (48.5–106.3)
Without blood			
1–2 d	154.17 (89.31–397.59)	3 (2–4)	475.7 (250.6–1,234.3)
3–4 d	14.15 (6.80–73.32)	10 (8–12)	139.0 (68.7–739.7)
>5 d	4.25 (2.25–13.36)	43 (40–46)	183.4 (97.9–578.1)
Overall		100	1001 (664–2,251)
STEC in South Australia			
With blood			
1–2 d	13.02 (9.50–18.37)	0	0
3–4 d	3.13 (2.36–4.45)	18 (6–30)	51.6 (16.2–101.2)
>5 d	2.02 (1.47–3.04)	68 (50–87)	123.5 (74.9–212.9)
Without blood			
1–2 d	157.18 (61.67–218.75)	3 (1–5)	432.5 (142.4–1,220.1)
3–4 d	14.35 (7.38–64.34)	6(1–11)	78.0 (13.6–400.1)
>5 d	4.31 (2.27–13.44)	6 (1–11)	2-3.0 (2.67–91.7)
Overall	13.02 (9.50–18.37)	100	815 (330–7,514)

*STEC, Shiga toxin–producing *Esherichia coli*. †Number of cases in the community for every notification. ‡Product of previous 2 columns.

The multipliers for *Salmonella, Campylobacter,* and STEC infections are thus 7 (95% CrI 4–16), 10 (95% CrI 7–22), and 8 (95% CrI 3–75), respectively. This indicates that overall, including mild to severe illness, ≈85% of salmonellosis cases, 90% of campylobacteriosis cases, and 88% of STEC cases are not reported.

### Community Incidence of *Salmonella, Campylobacter,* and STEC Infections

In the 5 years from 2000 to 2004, the national notifications for *Salmonella* ranged from 6,196 to 7,829 each year. The notifications from all states, except New South Wales, for *Campylobacter* ranged from 13,665 to 15,622. The notifications for STEC in the state of South Australia ranged from 27 to 39 from 2001 through 2004 (Table 2). The number of yearly reported cases was used to estimate the mean and standard deviation and then data were simulated from a normal distribution to give distributions of average “yearly notification number.” The product of the yearly notification number and the pathogen multipliers resulted in an estimate of the number of annual community infections of 49,843 (95% CrI 28,466–118,518) cases of salmonellosis, 224,972 (95% CrI 143,771–507,334) cases of campylobacteriosis, and 4,420 (95% CrI 2,407–10,196) cases of STEC infection. The corresponding estimates of annual incidence per 100,000 population are salmonellosis, 262 (95% CrI, 150–624); campylobacteriosis, 1,184 (95% CrI 756–2,670); and STEC infections, 23 (95% CrI 13–54).

## Discussion

In this study, we were able to provide CrIs of annual community incidence of 3 important infections from surveillance data. We used a method for determining pathogen-specific underreporting factors in Australia that has been deduced without the need to collect costly new data and includes an estimation of precision. This method is applicable to diseases other than infectious diarrhea, provided data on the components of the notification fraction are available: the proportion of case-patients who visit a doctor, the proportion who have a laboratory test, the sensitivity of the test, and the completeness of reporting of illness to surveillance. Even if collecting some additional data is necessary to estimate certain components of the notification fraction, this collecting may be worthwhile to obtain the added insight into the effects of particular diseases. Although knowing the incidence may not be necessary for detecting outbreaks and monitoring increased or decreased disease over time, this information is vitally important to policy makers. We consider it most important to furnish estimates with a measure of their precision and have used a simple simulation technique that is easily applied. If information is to be used in public health policy making, the responsible interpretation of results involves a realistic appreciation of potential error. Simple point estimates may give a misleading picture of certainty; the estimates of the community incidence of these foodborne diseases show a high degree of uncertainty that should be acknowledged when comparing estimates from other countries or times.

If this level of uncertainty is also found in other countries, then our confidence in apparent differences may be compromised. However, some differences appear to be so large that they are of interest nonetheless. When compared with multipliers for enteric diseases in other countries, Australia’s estimates were most similar to the estimates in the United Kingdom that were derived from a cohort study. It was estimated that for every case reported to surveillance, 3.2 cases of salmonellosis and 7.6 cases of campylobacteriosis existed in the community (*5*). However, the multiplier for salmonellosis in the United States has been estimated in the past at 39 (*3*,*19*), and the same factor was estimated in a recent US study (*6*). This recent study estimated the component probabilities of the notification fraction by using data from a population survey of diarrhea from 1996 in which the proportion of case-patients who visited a doctor was 12%. More recent surveys in the United States have put this estimate at ≈20% (*20*). If 20% is now more appropriate, then the US multiplier would reduce to ≈25. In the US study, blood in stool was found to be highly influential on stool test ordering by doctors (100% vs. 18% requested stool tests, depending on blood in stool) and not so influential on the probability of a case-patient visiting a doctor (15% vs. 12%), which was similar to our Australian study findings. The US study did not report duration as a predictor of visiting a doctor and of having a stool test. Because salmonellosis frequently lasts >5 days, adjusting for duration had a marked impact on our multiplier, reducing it considerably. If a similar influence of duration on visiting a doctor and ordering stool tests exists in the United States, and the symptom profile of salmonellosis is similar in the 2 countries, then the US multiplier would likely be further reduced.

The choice of the case definition of diarrheal illness used in population studies may also have affected calculations for the multipliers when the method of component probabilities was used. If the case definition itself includes features of severity that are predictors of visiting a doctor or of having a stool test, this may affect the proportion of cases that undergo these steps, thereby affecting the component probabilities used to calculate the multiplier. The laboratory sensitivity testing that provided another component probability may also affect the calculation of the multipliers. The quality assurance testing mimics some of the transport issues that occur in real life, but it probably represent a “best cases scenario” in which a patient sheds microorganisms at the time of collection, and good transport methods are available.

In addition to unavoidable uncertainty due to paucity of data, methodologic differences in each study, combined with differences in surveillance systems, can make international comparisons of disease incidence difficult. However, applying the multiplier for each country leads to an estimate of community incidence that is likely to make comparisons more meaningful than simply comparing notification rates. The notification rates of salmonellosis in each country are currently ≈70/100,000 population for the United Kingdom (*5*), 38/100,000 for Australia, and 12/100,000 (*21*) for the United States. Applying the underreporting factors of 3, 39, and 7 for the United Kingdom, United States, and Australia gives estimates of annual community incidence of ≈220/100,000, ≈470/100,000, and ≈262/100,000 (95% CrI 150–624), respectively. If more recent estimates of the proportion of case-patients who visit a doctor are used to calculate the underreporting factor in the United States, and a factor of 25 were applied to the number of notifications, the US rate becomes 300/100,000.

To validate results or assess the potential degree of error, another useful approach is to use differing methods and compare results in the same country. In Australia, 1 other possible method is to use results from an Australian cohort study of diarrheal disease (*22*). The study was a randomized controlled trial, conducted for 18 months, that assessed the health impact of water quality and treatment in Melbourne, Victoria, in 1999. Of 795 stool samples tested, 9 cases of salmonellosis (0.003 per person-year) and 24 cases of campylobacteriosis (0.007 per person-year) were identified. When these data are extrapolated to the notifications in Victoria, the community-to-notification ratios are 12.6 for salmonellosis and 9.3 for campylobacteriosis, which are comparable to our estimated multipliers of 7 (95% CrI 4–16) for salmonellosis and 10 (95% CrI 7–22) for campylobacteriosis.

Assessment of the functionality of surveillance is likely to lead to more effective control of disease in the community, and multipliers are 1 measure of the quality of surveillance systems. The relatively low ratio between reported enteric cases and the number of community cases in Australia suggests that the surveillance system is working reasonably effectively and therefore is likely to detect outbreaks.

This study provides an estimate of the community incidence of 3 important foodborne diseases in Australia. Such estimates are important in public health to assess the economic and human cost of these diseases and to help set priorities. The estimates in this study show that salmonellosis, campylobacteriosis, and STEC have considerable effects in the community, most of which go unreported. Calculation of multipliers for other diseases would also be worthwhile to inform public health practice.

## Supplementary Material

Technical Appendix Estimating Community Incidence of Salmonella, Campylobacter, and Shiga Toxin-producing Escherichia coli Infections, Australia
