# Supplementary material for: Estimating Community Incidence of Salmonella, Campylobacter, and Shiga Toxin–producing Escherichia coli Infections, Australia
Source: Emerg Infect Dis. 2008 Oct;14(10):1601–9. doi: 10.3201/eid1410.071042 (PMC2609882; doi:10.3201/eid1410.071042)
Supplement: Technical Appendix — Estimating Community Incidence of Salmonella, Campylobacter, and Shiga Toxin-producing Escherichia coli Infections, Australia [file 07-1042_Techapp-s1.pdf]

Estimating Community Incidence of *Salmonella, Campylobacter*, and Shiga Toxin–producing *Escherichia coli* Infections, Australia

Technical Appendix

The parameters for the simulated normal distributions (N: mean and SD) are shown in the following tables, along with the sources of the informative data outlined in Table 1 in the article's main text. Technical Appendix Table 1 shows the parameters for simulation of the distributions of the component probabilities and relates to Table 2 in the main text. Technical Appendix Table 2 shows the resultant multipliers and the parameters for the simulation of the distributions of the "percentage of notified cases by severity category" and relates to Table 3 in the main text.

The yearly notifications for *Salmonella, Campylobacter,* and STEC are shown in Table 4 in the main text and distributions were simulated using normal (mean 7157, SD 651), normal (mean 15,104, SD 946), and normal (mean 33.3, SD 5.67), respectively.

To assess the adequacy of using normal distributions compared with other distributions, simulations were also done using beta distributions when the informative data suggested this might be indicated. This applied to component probabilities close to 0 or 1, and to the yearly notification numbers. The final outcomes were similar for both methods of simulation as shown in online Technical Appendix Table 3. Because the outcomes were similar, and it is simpler to use a normal distribution, we applied this approach in all simulations described in the main text.

Technical Appendix Table 1. Parameters for simulation of the normal distributions [N: mean, sd] and sources of informative data for component probabilities of symptom multipliers for salmonellosis, campylobacteriosis, and STEC infections*

Condition/predictor symptoms	Component probabilities: a) Case-patient in the community visiting doctor,† proportion (95% CI), simulation [N: mean, SD]	b) Case-patient having stool tested,‡ proportion (95% CrI), simulation [N: mean, SD]	c) Positive stool results,§ proportion (95% CrI), simulation [N: mean, SD]	d) Notification by laboratory¶	Probability for case to be notified (a × b × c × d), median (95% CrI)	Symptom multiplier (inverse of NF), median (95% CrI)
Salmonellosis, duration, d						
With blood						
1–2	0.10 (0.07, 0.14) [N: 0.10, 0.015]	0.85 (0.72,0.98) [N: 0.85, 0.06]	0.98 (0.95,1.00) [N: 0.975, 0.012]	1.00	0.09 (0.06, 0.12)	11.39 (8.49, 16.36)
3–4	0.43 (0.31, 0.54) [N: 0.43, 0.06]	0.85 (0.72,0.98) [N: 0.85, 0.06]	0.98 (0.95,1.00) [N: 0.975, 0.012]	1.00	0.36 (0.25, 0.46)	2.82 (2.17, 3.98)
>5	0.67 (0.46, 0.88) [N: 0.67, 0.11]	0.85 (0.72,0.98) [N: 0.85, 0.06]	0.98 (0.95,1.00) [N: 0.975, 0.012]	1.00	0.55 (0.368, 0.75)	1.81 (1.33, 2.72)
Without blood						
1–2	0.10 (0.07, 0.14) [N: 0.10, 0.015]	0.07 (0.02, 0.12) [N: 0.07, 0.02]	0.98 (0.95,1.00) [N: 0.975, 0.012]	1.00	0.01 (0.003, 0.01)	143.29 (83.30, 371.0)
3–4	0.43 (0.31, 0.54) [N: 0.43, 0.06]	0.19 (0.071,0.36) [N: 0.19, 0.08]	0.98 (0.95,1.00) N(0.975, 0.012]	1.00	0.08 (0.010, 0.16)	13.06 (6.37, 67.83)
>5	0.67 (0.46, 0.88) [N: 0.67, 0.11]	0.40(0.133,0.67) [N: 0.40, 0.14]	0.98 (0.95,1.00) [N: 0.975, 0.012]	1.00	0.25 (0.075, 0.48)	3.93 (2.10, 11.92)
Campylobacteriosis, duration, d						
With blood						
1–2	0.10 (0.07, 0.14) [N: 0.10, 0.015]	0.85 (0.72,0.98) [N: 0.85, 0.06]	0.90 (0.85, 0.95) [N: 0.9, 0.02]	1.00	0.08 (0.056, 0.11)	12.40 (9.16, 17.82)
3–4	0.43 (0.31, 0.54) [N: 0.43, 0.06]	0.85 (0.72,0.98) [N: 0.85, 0.06]	0.90 (0.85, 0.95) [N: 0.9, 0.02]	1.00	0.33 (0.231, 0.43)	3.06 (2.32, 4.33)
>5	0.67 (0.46, 0.88) [N: 0.67, 0.11v	0.85 (0.72,0.98) [N: 0.85, 0.06]	0.90 (0.85, 0.95) [N: 0.9, 0.02]	1.00	0.51 (0.339, 0.70)	1.97 (1.42, 2.95)
Without blood						
1–2	0.10 (0.07, 0.14) [N: 0.10, 0.015]	0.07 (0.02, 0.12) [N: 0.07, 0.02]	0.90 (0.85, 0.95) [N: 0.9, 0.02]	1.00	0.01 (0.002, 0.01)	154.17 (89.31, 397.59)
3–4	0.43 (0.31, 0.54) [N: 0.43, 0.06]	0.19 (0.071,0.36) [N: 0.19, 0.08]	0.90 (0.85, 0.95) [N: 0.9, 0.02]	1.00	0.07 (0.009, 0.15)	14.15 (6.80, 73.32)
>5	0.67 (0.46, 0.88) [N: 0.67, 0.11]	0.40(0.133,0.67) [N: 0.40, 0.14]	0.90 (0.85, 0.95) [N: 0.9, 0.02]	1.00	0.24 (0.068, 0.44)	4.25 (2.25, 13.36)
STEC in South Australia, duration, d						
With blood						
1–2	0.10 (0.07, 0.14) [N: 0.10, 0.015]	0.85 (0.72,0.98) [N: 0.85, 0.06]	0.88 (0.83, 0.93) [N: 0.975, 0.012] x [N: 0.90, 0.023]	1.00	0.08 (0.0.05, 0.11)	13.02 (9.50, 18.37)
3–4	0.43 (0.31, 0.54) [N: 0.43, 0.06]	0.85 (0.72,0.98) [N: 0.85, 0.06]	0.88 (0.83, 0.93) [N: 0.975, 0.012] x [N: 0.90, 0.023]	1.00	0.32 (0.22, 0.42)	3.13 (2.36, 4.45)

>5	0.67 (0.46, 0.88) [N: 0.67, 0.11]	0.85 (0.72,0.98) [N: 0.85, 0.06]	0.88 (0.83, 0.93) [N: 0.975, 0.012] x [N: 0.90, 0.023]	1.00	0.50 (0.33, 0.68)	2.02 (1.47, 3.04)
Without blood						
1–2	0.10 (0.07, 0.14) [N: 0.10, 0.015]	0.07 (0.02, 0.12) [N: 0.07, 0.02]	0.88 (0.83, 0.93) [N: 0.975, 0.012] x [N: 0.90, 0.023]	1.00	0.01 (0.001, 0.02)	157.18 (61.67, 218.75)
3–4	0.43 (0.31, 0.54) [N: 0.43, 0.06]	0.19 (0.071,0.36) [N: 0.19, 0.08]	0.88 (0.83, 0.93) [N: 0.975, 0.012] x [N: 0.90, 0.023]	1.00	0.07 (0.01, 0.14)	14.35 (7.38, 64.34)
>5	0.67 (0.46, 0.88) [N: 0.67, 0.11]	0.40(0.133,0.67) [N: 0.40, 0.14]	0.88 (0.83, 0.93) [N: 0.975, 0.012] x [N: 0.90, 0.023]	1.00	0.23 (0.07, 0.44)	4.31 (2.27, 13.44)

*Crl, credible interval; NGS, National Gastroenteritis Survey; STEC, Shiga toxin–producing *Escherichia coli*.
†Informative data from NGS.
‡Informative data from NGS (for no blood in stool) and GP surveys (for blood in stool).
§Informative data from quality assurance testing.
¶Informative data from OzFoodNet Constant.

Technical Appendix Table 2. Parameters for simulation of the normal distributions [N: mean, sd] for the symptom categories for salmonellosis, campylobacteriosis and STEC infections*

Condition/severity category	a) Multipliers (cases in the community for every notification; from Table 1), median (95% CrI)	b) No. out of 100 notified cases in symptom category,† % (95% CrI) [N: mean, SD]	No. cases in the community for every 100 notified (a × b), median (95% CrI)
Salmonellosis, duration, d			
With blood			
1–2	11.39 (8.49–16.36)	1 (0– 3) [N: 1, 0.8]	12.7 (0.8–32.1)
3–4	2.82 (2.17– 3.98)	7 (5–10) [N: 7,1.2]	19.9(12.6–30.9)
≥5	1.81 (1.33–2.72)	42 (37–47) [N: 42, 2.2]	76.6(54.3–116.0)
Without blood			
1–2	143.29 (83.30–371.0)	2 (1–4) [N: 2, 0.8]	282.6 (50.4–870.3)
3–4	13.06 (6.37– 67.83)	7 (5–10) [N: 7,1.2]	91.8 (40.3–533.5)
>5	3.93 (2.10–11.92)	41 (36–46) [N: 41, 2.2]	160.8 (85.8–513.8)
Overall		100	695 (399–1,643)
Campylobacteriosis. duration, d			
With blood			
1–2	12.40 (9.16–17.82)	2 (1–3) [N: 2, 0.25]	24.8 (16.3–38.6)
3–4	3.06 (2.32–4.33)	8 (6–10) [N: 8, 1]	24.3 (15.8–36.9)
≥5	1.97 (1.42–2.95)	34 (31–37) [N: 34, 1.5v	67.92 (48.5–106.3)
Without blood			
1–2	154.17 (89.31–397.59)	3 (2–4) [N: 3, 0.5]	475.7 (250.6–1234.3)
3–4	14.15 (6.80–73.32)	10 (8–12) [N: 10, 1]	139.0 (68.7–739.7)
≥5	4.25 (2.25–13.36)	43 (40–46) [N: 43, 1.5]	183.4 (97.9–578.1)
Overall		100	1001 (664, 2251)
STEC in South Australia, duration, d			
With blood			
1–2	13.02 (9.50–18.37)	0	0
3–4	3.13 (2.36–4.45)	18 (6–30) [N: 18, 6]	51.6 (16.2–101.2)
≥5	2.02 (1.47–3.04)	68 (50–87) [N: 68, 7]	123.5 (74.9–212.9)
Without blood			
1–2	157.18 (61.67–218.75)	3 (1– 5) [N: 3, 1.5]	432.5 (142.4–1,220.1)
3–4	14.35 (7.38–64.34)	6(1– 11) [N: 6, 2.5]	78.0 (13.6–400.1)
≥5	4.31 (2.27–13.44)	6 (1–11) [N: 6, 2.5]	23.0 (2.67– 91.7)
Overall	13.02 (9.50–18.37)	100	815 (330–7,514)

*CrI, credible interval; STEC, Shiga toxin–producing *Escherichia coli*.
†Informative data from case control studies.

Technical Appendix Table 3. Comparison of estimates of annual number of cases in the community and incidence per 100,000 persons in Australia, using a) normal distributions for all simulations and using b) beta distributions where indicated

Pathogen	No. community cases for every 100 notified, median (95% CrI)	Estimated annual no. community cases in Australia, median (95% CrI)	Estimated community incidence in Australia, per 100,000 persons, median (95% CrI)
Salmonella			
a) Simulation with all normal distributions	695 (399–1,643)	49,843 (28,466–118,518)	262 (150–624)
b) Simulation with beta distributions where indicated*	674 (394–14,945)	48,477 (27,993–105,338)	255 (147–554)
Campylobacter			
a) Simulation with all normal distributions	10 (6.6–22.5)	224,972 (143,771–507,234)	1,184 (756–2,670)
b) Simulation with beta distributions where indicated*	10 (5.4–24.9)	225,003 (115,484–568,268)	1,184 (608–2,991)
STEC			
a) Simulation with all normal distributions	8 (3–75)	4,420 (2,407–10,196)	23 (13–54)
b) Simulation with beta distributions where indicated*	9 (4–21)	4,418 (2,276–11,159)	23 (12– 59)

*Beta distributions used for simulations where informative data suggested probabilities close to 0 or 1 or other nonsymmetric distributions.
